# Colorectal Cancer Racial Equity Post Volume, Content, and Exposure: Observational Study Using Twitter Data

**DOI:** 10.2196/63864

**Published:** 2025-02-03

**Authors:** Chau Tong, Drew Margolin, Jeff Niederdeppe, Rumi Chunara, Jiawei Liu, Lea Jih-Vieira, Andy J King

**Affiliations:** 1 School of Journalism University of Missouri Columbia, MO United States; 2 Institute for Data Science and Informatics University of Missouri Columbia, MO United States; 3 Department of Communication Cornell University Ithaca, NY United States; 4 Jeb E. Brooks School of Public Policy Cornell University Ithaca, NY United States; 5 Department of Biostatistics New York University New York, NY United States; 6 Department of Computer Science & Engineering New York University New York, NY United States; 7 STEM Translational Communication Center College of Journalism and Communications University of Florida Gainesville, FL United States; 8 Department of Advertising College of Journalism and Communications University of Florida Gainesville, FL United States; 9 School of Engineering and Applied Science University of Virginia Charlottesville, VA United States; 10 Cancer Control & Population Sciences Huntsman Cancer Institute Salt Lake, UT United States; 11 Department of Communication University of Utah Salt Lake, UT United States

**Keywords:** racial equity information, information exposure, health disparities, colorectal cancer, cancer communication, Twitter, X

## Abstract

**Background:**

Racial inequity in health outcomes, particularly in colorectal cancer (CRC), remains one of the most pressing issues in cancer communication and public health. Social media platforms like Twitter (now X) provide opportunities to disseminate health equity information widely, yet little is known about the availability, content, and reach of racial health equity information related to CRC on these platforms. Addressing this gap is essential to leveraging social media for equitable health communication.

**Objective:**

This study aims to analyze the volume, content, and exposure of CRC racial health equity tweets from identified CRC equity disseminator accounts on Twitter. These accounts were defined as those actively sharing information related to racial equity in CRC outcomes. By examining the behavior and impact of these disseminators, this study provides insights into how health equity content is shared and received on social media.

**Methods:**

We identified accounts that posted CRC-related content on Twitter between 2019 and 2021. Accounts were classified as CRC equity disseminators (n=798) if they followed at least 2 CRC racial equity organization accounts. We analyzed the volume and content of racial equity–related CRC tweets (n=1134) from these accounts and categorized them by account type (experts vs nonexperts). Additionally, we evaluated exposure by analyzing follower reach (n=6,266,269) and the role of broker accounts—accounts serving as unique sources of CRC racial equity information to their followers.

**Results:**

Among 19,559 tweets posted by 798 CRC equity disseminators, only 5.8% (n=1134) mentioned racially and ethnically minoritized groups. Most of these tweets (641/1134, 57%) addressed disparities in outcomes, while fewer emphasized actionable content, such as symptoms (11/1134, 1%) or screening procedures (159/1134, 14%). Expert accounts (n=479; 716 tweets) were more likely to post CRC equity tweets compared with nonexpert accounts (n=319; 418 tweets). Broker accounts (n=500), or those with a substantial portion of followers relying on them for equity-related information, demonstrated the highest capacity for exposing followers to CRC equity content, thereby extending the reach of these critical messages to underserved communities.

**Conclusions:**

This study emphasizes the critical roles played by expert and broker accounts in disseminating CRC racial equity information on social media. Despite the limited volume of equity-focused content, broker accounts were crucial in reaching otherwise unexposed audiences. Public health practitioners should focus on encouraging equity disseminators to share more actionable information, such as symptoms and screening benefits, and implement measures to amplify the reach of such content on social media. Strengthening these efforts could help bridge disparities in cancer outcomes among racially minoritized groups.

## Introduction

Colorectal cancer (CRC) is among the most common and deadly cancers in the United States [1]. Although there have been advancements in CRC prevention and treatment, racial disparities in CRC morbidity and mortality persist, with Black Americans facing higher CRC risks [2]. Thus, promoting CRC awareness and screening behaviors among Black Americans for CRC prevention and early detection to reduce racial disparities has become an urgent task for CRC-related health communication and education [3-9]. Improving the quality of information about CRC prevention, detection, and advocacy is an important step in providing education and resources for improved decision-making to contribute to efforts to reduce CRC disparities among Black Americans [10-14].

Web-based sources—such as social media and related platforms—are an increasing source of health information for the general public, particularly for Black Americans. Black Americans are more likely than White Americans to rely on a wide variety of sources to acquire CRC screening-specific information—including web-based sources [15-20]. Research on CRC information has generally focused on the extent to which information presented is misleading, distracting, or inaccurate. For example, researchers found that medical professionals classified almost 40% of CRC YouTube content as “not useful,” and such content was viewed and engaged with more frequently than higher-quality content [17]. TikTok content varied in its utility and accuracy, but researchers commonly identified inaccurate and misleading content [18]. On the other hand, other research on Twitter (now X) indicated that tweets about CRC were mostly accurate [19], while other studies noted that CRC received less Twitter attention than other (eg, breast and prostate) cancer sites [20].

For cancer topics where there are disparities by race, ethnicity, or other identities, misleading information is not the only way that members of the public can be underserved. Specifically, if specific information relevant to a particular community does not reach this audience [14,21-23], the information can be “misleading” in another sense. For example, since the rate of mortality from CRC among Black Americans is higher compared with other racial groups, misperceptions prevail as to the recommended screening age for this group, even more so after the official guideline changes from the recommended screening age of 50 to 45 years [16]. Thus, if Black Americans are only exposed to messages that describe overall population averages or recommendations, they may be inadvertently “misled” in the sense that they are not given access to important information to inform their decisions about prevention and screening [14,22].

This study focuses on an aspect of this problem of specialized/targeted information reaching populations that experience health inequities. Specifically, we examine the extent to which messages sent about and related to inequities regarding CRC by those motivated to address the issue are likely to reach historically marginalized populations or whether they tend to remain within the professional circles of the health experts themselves. The basic problem is that while in theory, social media can connect anyone to anyone, in practice, people tend to connect with similar others [24], resulting in clusters of tight-knit groups with overlapping, shared relationships [25]. These structures, sometimes referred to as “echo chambers,” can create a particular problem for the dissemination of so-called “expert” information that, by definition, comes from specialized sources like medical journals and scholarly publications. For example, if a particular group is underrepresented in the scientific community, and the latest expert findings regarding the structural barriers to treatment and elevated CRC mortality faced by this underrepresented group are shared only among a cluster of experts, this important information may not reach and inform prevention, screening, and treatment decisions for people with historically minoritized identities.

Our study attempts to understand this distinction—between what is said about racial equity and CRC by accounts that are concerned with it and what is shared with populations facing these health outcome inequities—by analyzing both the volume and content of racial equity–related tweets from CRC equity disseminator accounts—an original concept that we introduced, defined as Twitter accounts that disseminate information related to racial equity concerning CRC outcomes.

Some individuals break out of communicating only within smaller, isolated clusters. Social network theory refers to these individuals as brokers [26,27]. Brokers are connected to different communities and are thus a key source of information transfer and diffusion [25,26]. They thus have an outsized influence on the information exposure for individuals and groups who are not connected to experts or other specialized communities. In essence, those who are disconnected do not hear everything that is said, but they hear what the brokers say. For this reason, this study also addresses a gap in the literature on CRC social media content and exposure by examining the influence of broker accounts—a special subset of CRC equity disseminator accounts that is more likely to reach unique audiences in disseminating racial equity content.

By addressing both aspects of content and dissemination, this study contributes to a more nuanced understanding of the public communication environment surrounding CRC racial equity, which will help inform future health communication and education efforts aimed at reducing CRC disparities.

## Methods

### Identifying CRC Equity Organization Accounts

**Figure 1:**
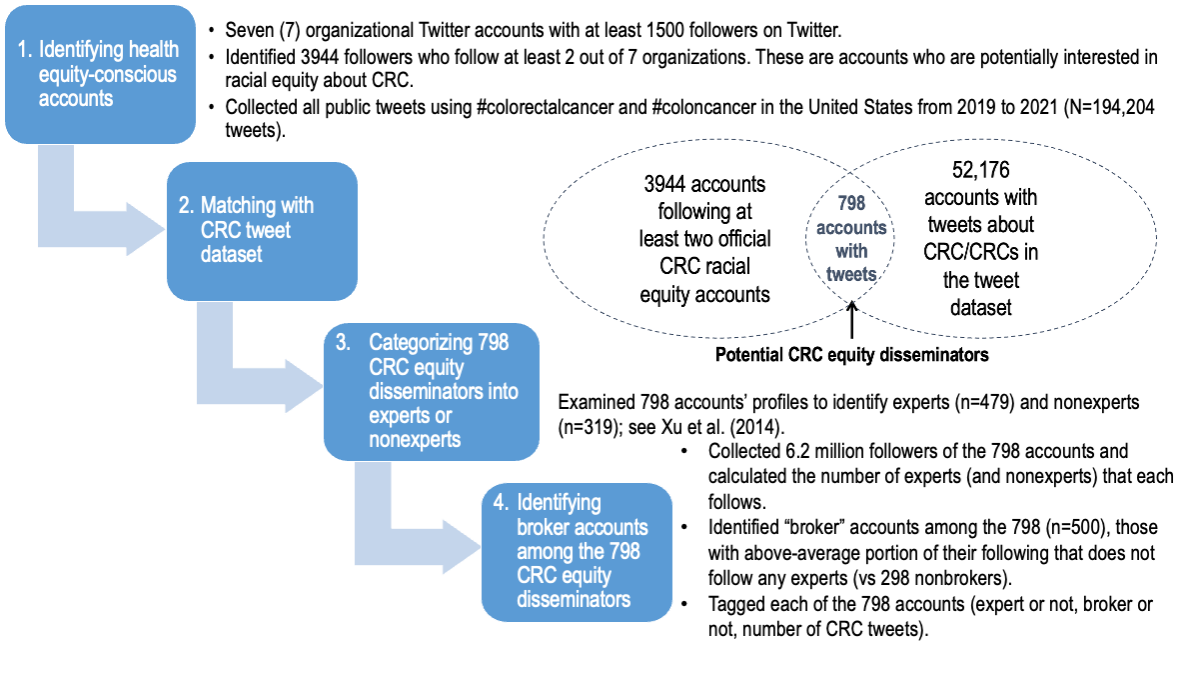
Workflow for mapping CRC equity networks and roles. CRC: colorectal cancer.

We first identified a list of organizations whose primary focus is health equity or the health of racially and ethnically minoritized populations and the promotion of preventive health behaviors (including CRC screening; Figure 1). We used the US Department of Health and Human Services’ National Minority Organizations list [28] to identify these health agencies (such as the National Institute on Minority Health and Health Disparities) and supplemented the list with organizations specifically dedicated to CRC prevention (such as the Association of Black Gastroenterologists and Hepatologists and the Colorectal Cancer Alliance). We then looked for the corresponding public Twitter accounts of these organizations and narrowed our pool from 18 to 7 accounts that had at least 1500 followers. The average number of followers was 9204, with the National Institute on Minority Health and Health Disparities being the most followed account (27,451 followers) and the Center for Black Health being the least followed (1594 followers). We retrieved the list of followers of these 7 organization accounts, resulting in a total of 59,669 unique followers (average number of organizations followed=1.08, SD 0.32). The majority of these users (n=55,725, 93.4%) only followed 1 of the 7 health equity organizations. A much smaller proportion, 3944 (6.6%) users followed at least 2 of the 7 organizations and were considered as having a potential interest in the topic of racial equity regarding CRC.

### Identifying CRC Equity Disseminators

We collected public tweets about CRC from 2019 to 2021 using the keywords “coloncancer,” “colorectalcancer,” “colon cancer,” and “colorectal cancer” and restricted the region to the United States (N=194,204 tweets) due to possible country-related differences about CRC advocacy and screening recommendations (eg, free screening services in some countries). Of the 3944 users identified as having a potential interest in racial equity about CRC, there were 798 accounts that produced tweets containing any of the terms above (n=25,093 CRC-related tweets; 19,559 of them are unique). We considered these 798 accounts as potential CRC equity disseminators; those who could play a crucial role in disseminating information about CRC racial equity to people who may not follow equity organizations. We note that only 0.7% of equity disseminators’ followers also followed any CRC equity organization account, suggesting that these users’ exposure to CRC racial equity content is less likely to come from equity organizations but more likely to come from equity disseminators themselves.

### Identifying Expert and Nonexpert Accounts Among the CRC Equity Disseminators

To identify the identities of the 798 CRC equity disseminators, we categorized Twitter users based on their self-provided Twitter profiles. Following the inductive categorization framework introduced by Xu et al [29] and their typology for health knowledge sharing in a Twitter-based community of practice, in which users were grouped by their degree of health care involvement (ie, expert, those with experience, those interested in health topics, and the general public) and their health care roles (ie, providers, advocacy, engaged or average consumers, media, government, and non–health care–related organizations), we classified each of our 798 as either “expert” (including professional health service providers such as health care practitioners, health scientists, organizations, research laboratories, and medical centers) or “non-expert” (including advocacy groups, organizations, or individuals; average or engaged consumers; media; and government agencies; see Xu et al [29] for details on each category and definition). This resulted in 479 experts versus 319 nonexperts.

### Identifying Broker and Nonbroker Accounts Among the CRC Equity Disseminators

In addition to tagging each of the 798 accounts as either an expert or nonexpert account, we also determined whether each was a broker or nonbroker. To arrive at this categorization, we collected all the followers of the 798 accounts (N=6,266,269 unique followers) and calculated the number of expert and nonexpert accounts each of the followers followed. An account was classified as a broker account if more than half of its followers did not follow any experts (other than this one if the account in focus itself was an expert account). This resulted in 500 brokers and 298 nonbrokers.

### Intersection of Expertise and Brokerage: The Role of Expert Brokers

Now that each of the 798 CRC equity disseminator accounts is tagged as either an expert or not and a broker or not, an account was considered an expert-broker account if it fell into both categories. Conceptually, an account is an expert-broker if it is an expert account, and more than half of its followers do not follow any other experts. Based on this definition, among the 479 experts, 254 (53%) were expert-brokers. These expert-brokers are especially important because they can uniquely connect people to content produced by experts. For example, a doctor who is followed by accounts that do not follow other doctors is more likely to be a crucial unique source of information for their followers who would not get important information (eg, about screening recommendations) elsewhere. Because our study is about access to medical information pertinent to minoritized populations, we are interested in the role of these expert-broker accounts as opposed to expert-nonbrokers (ie, whose followers tend to follow other experts as well, in this case, a doctor whose followers are mostly individuals who also follow many other doctors).

### Coding of CRC Equity Tweets

We are interested in the specific CRC content about racial equity sent by the 798 CRC equity disseminators. We identified these tweets using a combination of (computational) dictionary tagging and manual (human) coding. We first tagged CRC-related tweets using a custom dictionary consisting of terms and phrases signaling historically minoritized racial groups (see full list of these terms and phrases in Table 1). Several specific terms were adapted from previous research such as [30,31].

Next, we analyzed the major themes in these CRC racial equity tweets and inductively identified 13 nonmutually exclusive categories for manual coding (Table 2). Two coders went through the codebook training process and reliably coded 10% of random tweets into 13 content subcategories (Krippendorf α ranged from 0.85 to 0.94). The subcategories are not mutually exclusive, so that a given tweet may fall into multiple categories. In Table 2, examples of tweets, in line with best practices of social media research [32], we have removed the names associated with certain hashtags and mentions to ensure the tweets are anonymized and cannot be traced back to their original authors.

**Table 1:** Keywords for retrieving potential racial equity content from colorectal cancer tweets.

Categories	Keywords
People of color	“people of color,” “community of color,” “communities of color,” “colored,” “poc,” “woc,” “woman of color,” “minority,” “minorities,” “racial disparity,” “racial disparities,” and “racism”
Black	“black*,” “african american*,” “af american,” “brown,” “blk,” “blm,” “brother,” “brotha,” “m4bl,” “sister,” and “sista”
Hispanic/Latino/a/x	“hispanic*,” “latino*,” “latinx*,” and “latina*”
Indigenous	“indigenous,” “american indian*,” and “native american*”
Asian	“asian*” and “asian american*”

**Table 2:** Categories, definitions, examples of colorectal cancer (CRC) racial equity tweets, and intercoder agreement.

Category	Definition	Examples of tweets	Krippendorf α
Outcome disparity	Mention of information about CRC or CRCs specific to more than 1 race or ethnicity; comparison/contrast between groups	Murphy et al show black/white disparity in rise in incidence driven by rectal cancer. Any data on Hispanics and Asians? Research presented at #[national research conference] shed some light on disparities in outcomes for African American and Caucasian #colorectalcancer patients. Listen to the story from @[media outlet]:	0.92
Call to action	Suggesting people talk to their doctors about screening; encouraging people to get screened as recommended by their doctors; reminding people to stay up to date on screening, etc.	Black men & women manifest at an earlier age with #colorectalcancer & should start screening at 40-45yo. If you have not had colonoscopy, please talk to your PCP. @FightCRC @CCAlliance #disparities Let’s change the stats. Help us prevent #cancer by encouraging brothers (ages 45-75) in Utah to visit #[national research conference] #CuttingCRC #MinorityHealth #BlackHistoryMonth	0.94
Risk factors	Mention of information of risks that are associated with CRC, such as family history, physical inactivity, diet, alcohol use, lynch syndrome, being overweight, being older, etc.	What are some preventive measures can #Latinos and all people take to lower their #colorectalcancer risk? #coloncancerawarenessmonth Get a colonoscopy if you smoke or if you are a Black male or if there is cancer in your family.	0.89
Symptoms	Mention of known indicators of CRC, such as blood in stool, changes in bowel habits, bloating, cramps, gas, pain, weight loss for no reason, etc.	On world cancer day 2021, I’m urging everyone to get screened for colorectal cancer. Esp. Black and brown folks. If you are 45 or older or are experiencing symptoms (abdominal pain, thin stool, blood in stool, fatigue, unexplained weight loss), talk to your doctor. A colonoscopy could save your life.	0.88
Raising awareness	Mention or stress the need of more widespread and open communication about CRC	Just like Black people need to have the talk with their sons about sex and being careful of cops, they need to be talking about colorectal cancer and how to prevent it. It is not just an old person’s disease.	0.85
Advocacy	Advocating or endorsing screening in general, or specific CRC screening options	African Americans have the highest incidence of #colorectalcancer and highest mortality rate of any racial or ethnic group, according to the @AmericanCancer Society. Get screened! If not for you, do it for your loved ones! #BlackHistoryMonth #coloncancer #cancer #colonoscopy Promote colonoscopy screening among low-income Latinos at average risk of #ColorectalCancer. Here’s new research using randomized clinical trial.	0.9
Celebrities’ stories	Individual stories concerning public figures who are patients, advocates, or survivors of CRC	In GQ, Author Ibram X. Kendi writes about his #diagnosis of #coloncancer at 35, the early #warningsigns of #CRC and his experience going thru #treatment. #Blackmen are 40% more likely to die of #colorectalcancer than other races. Kendi is now #cancerfree. Back on @[media outlet] to provide commentary on the stigma of colon cancer and the benefit of the @CCAlliance Buddy system. The segment focused on @[user], and her story of being diagnosed w/ colon cancer at 50.	0.94
Personal stories	Individual stories concerning regular people, patients, advocates related to CRC and CRC screening	An artist, an advocate, he gave my black boys a superhero with whom to identify. He reminds us #ColonCancer screening isn’t elegant but necessary and should start 5yrs earlier in Af Am patients #womenshealth #parenting #healthdisparities. [User], a staunch patient advocate and #colorectalcancer survivor, is out here raising much-needed awareness about a #cancer highly preventable in most, and all too common, esp in the Black community.	0.93
Mortality	Mention of the severity of CRC, mortality rates	March is Colorectal Cancer Awareness Month! Colon cancer is the second most common cancer among Indigenous people, and the second leading cause of cancer death. #GetBehindCRCScreening to help us end colon cancer in Indian Country! Unfortunately, colorectal cancer is becoming a killer of young Black men, which we can conquer only by talking openly about symptoms, the value of colonoscopy screening, and sharing our experiences.	0.9
Incidence of CRC	Mention of the prevalence of CRC and statistics of its impact	#ColorectalCancer is more common in men than women and among those of African American descent. The rate of new cases of colorectal cancer was 38.2 per 100,000 men and women per year based on 2013-2017 cases, age adjusted. Learn more from our page on #CRC. [User] discusses the concerning trends of #ColorectalCancer rates in younger patients and the higher incidence and lower survival rates being seen in Black people. #[local cancer center] #local cancer center].	0.89
Screening prevalence	Mention of the general prevalence of CRC screening or statistics about screening rates	Fewer than half of Native Americans over 50 are up-to-date with #ColorectalCancer screening. Learn how CDC is working with @[government agency] and @[government agency] to help. Due to COVID-19, colonoscopy screening for colon cancer among minorities declined nearly 90%. We are going #BlueForCRC to raise awareness and encourage preventative screenings.	0.89
Screening information	Providing details about screening options, or screening logistics	NEW ACG Clinical Guidelines on CRC Screening! info: -screen @ 45 (avg-risk adults) -recs if family Hx -qual indicators (ADR & withdrawal) -recs on aspirin for risk -1 versus 2-step tests -interventions to increase screening, esp. among African Americans “Mailed stool blood tests; followup phone calls are examples of effective strategies that increased #colorectalcancer screening among African Americans” @FightCRC	0.87
Benefits of screening	Using reasons to appeal to why screening is necessary, worth the effort, and beneficial	Getting routine screenings is imperative, regardless of family history. It’s worth it to have peace of mind that you don’t have colorectal cancer. #blackcommunity Colorectal cancer screening can detect cancer early—when it is most curable. Learn more about your screening options! #LatinxinMedicine	0.86

### Volume of Exposure to CRC Equity Tweets

We then examined the extent to which the followers of the 798 accounts were exposed to CRC equity tweets. Different from the analysis of content, which deals with unique tweets or unique equity tweets only (n=1134), the analysis of exposure deals with nonunique equity tweets (n=1333) to adjust for the cumulative effect of exposure to both unique tweets and retweets. As the exact mechanisms behind Twitter’s algorithms to display specific content to a user at a given time are unknown, we relied on Twitter’s public documentation [33], assuming that by subscribing to an account, a user could be exposed to all posts and updates from that account. Based on this logic, for each of the 6,266,269 unique followers of the 798 CRC potential disseminator accounts, we calculated the number of the accounts they followed, as well as the number of (nonunique) general CRC tweets and CRC equity tweets they could have been exposed to (which is equal to the number of the general CRC tweets and CRC equity tweets posted by the accounts they followed). Finally, we estimated the volume of exposure to CRC equity tweets among these followers overall and by account types (ie, whether the followed account is an expert, or a broker).

### Ethical Considerations

All analyses are based on publicly available data. To respect privacy, we do not disclose usernames and identifiable information in our paper and only report aggregate results.

## Results

### Volume and Content of CRC Racial Equity Tweets

We begin by analyzing the content produced by the 798 potential equity disseminator accounts. Of the 19,559 (unique) CRC tweets these accounts produced, only 5.8% (n=1134) mentioned a historically minoritized racial group and thus were deemed likely to be specifically about CRC racial equity, disparity, or racial identity–specific impact/information (although not all of them necessarily discussed racial information in ways that signaled fairness and justice).

Table 3 provides a summary of the tweet categories’ respective volumes and proportions. Notably, outcome disparity (ie, specifying disparities in CRC outcomes between racial and ethnic groups) was the most common type of content, appearing in 56.53% (641/1134) of unique CRC racial equity tweets. Other common content categories were a call to action (ie, encouraging CRC detection/prevention; (505/1134, 44.53%), CRC risks or risk factors (351/1134, 30.95%), and raising awareness of CRC (ie, emphasizing the need to communicate more about CRC; 337/1134, 29.72%). In contrast, details about CRC screening logistics or options (159/1134, 14.02%), benefits (98/1134, 8.64%), prevalence of screening (66/1134, 5.82%), and CRC symptoms (11/1134, 0.97%) were less common, though still present.

We next investigated which types of accounts tended to post different kinds of messages. Experts were more likely than nonexperts to send tweets about CRC racial equity, (*z*=14.88, *P*<.001). Specifically, of the 8210 unique CRC tweets produced by experts, 8.7% (n=716) of them mentioned at least 1 racially minoritized group. Nonexpert accounts, by contrast, only mentioned a racially minoritized group in 3.7% (418/11,349) of unique tweets they produced. There were also differences between expert and nonexpert accounts in their proportions of tweets for specific content subcategories. However, the only statistically significant difference is that experts focused more on celebrities’ stories than nonexperts (*P*=.04; Table 3).

**Table 3:** Volume and proportion of colorectal cancer (CRC) racial equity tweets by content and account type. Proportions of specific content subcategories are compared between expert versus nonexpert accounts, brokers versus nonbrokers, and expert-brokers versus expert-nonbrokers.

Content type	Number of unique racial equity tweets from CRC equity disseminators (n=1134 tweets), n (%)	Number of unique racial equity tweets from experts (n=716 tweets), n (%)	Number of unique racial equity tweets from nonexperts (n=418 tweets), n (%)	Number of unique racial equity tweets from brokers (n=518 tweets), n (%)	Number of unique racial equity tweets from nonbrokers (n=616 tweets), n (%)	Number of unique racial equity tweets from expert-nonbrokers (n=556 tweets), n (%)	Number of unique racial equity tweets from expert-brokers (n=160 tweets), n (%)
Outcome disparity	641 (56.53)	412 (57.54)	229 (54.78)	282 (54.44)	360 (58.44)	322 (57.91)	90 (56.25)
Call to action	505 (44.53)	312 (44.83)	184 (44.02)	229 (44.21)	276 (44.80)	252 (45.32)	69 (43.13)
Risk factors	351 (30.95)	227 (31.70)	124 (29.67	168 (32.43)	183 (29.70	160 (28.78)^a^	67 (41.88)^a^
Raising awareness	337 (29.72)	201 (28.07)	136 (32.54)	162 (31.27)	175 (28.41)	154 (27.70)	47 (29.38)
Advocacy	263 (23.19)	171 (22.91)	99 (23.68)	124 (23.94)	139 (22.56)	129 (23.20)	35 (21.88)
Celebrities’ stories	245 (21.60)	164 (23.88)^a^	74 (17.70)^a^	110 (21.24)	135 (21.92)	123 (22.12)^a^	48 (30.00)^a^
Mortality or death	194 (17.11)	130 (18.16)	64 (15.31)	83 (16.02)	111 (18.02)	97 (17.45)	33 (20.63)
Incidence of CRC	160 (14.11)	116 (13.97)	60 (14.35)	74 (14.29)	86 (13.96)	72 (12.95)	28 (17.50)
Screening information	159 (14.02)	100 (16.20)	43 (10.29)	70 (13.51)	89 (14.44)	80 (14.39)^a^	36 (22.50)^a^
Personal stories	125 (11.02)	75 (10.47)	50 (11.96)	69 (13.32)^a^	56 (9.09)^a^	54 (9.71)	21 (13.13)
Benefits of screening	98 (8.64)	61 (8.52)	37 (8.85)	52 (10.04)	46 (7.47)	42 (7.55)	19 (11.88)
Screening prevalence	66 (5.82)	39 (5.45)	27 (6.46)	27 (5.21)	39 (6.33)	34 (6.12)	5 (3.13)
Symptoms	11 (0.97)	9 (1.26)	2 (0.48)	4 (0.77)	7 (1.14)	7 (1.26)	2 (1.25)

^a^Values different at *P*<.05 level.

We also compared brokers to nonbrokers. Recall the definition of brokers as a subset of CRC equity disseminator accounts where more than half of their followers did not follow any experts (or any experts other than itself, if the account in focus was an expert account). Overall, brokers were less likely to talk about equity (518 tweets) than nonbrokers (616 tweets).

Examining the volume of equity tweets by expert brokers versus expert nonbrokers, we found that expert brokers produced fewer equity tweets (160 tweets) compared with expert nonbrokers (556 tweets). An examination at the account level revealed that only 30% (77/254) of experts who are brokers tweeted at least once about equity, compared with 46% (103/225) of expert-nonbrokers who did so. This pattern indicates that those who talk about equity more frequently are those with less reach beyond the community and who would otherwise not learn about this topic.

### Exposure to CRC Racial Equity Tweets

We now turn to the question of how different types of accounts impact the kind of information that their followers are exposed to. In other words, which of the 798 potential CRC equity disseminators do the “best” job of exposing their followers to equity content. Since both tweets and retweets (not just unique tweets) contribute to exposure, the outcome of interest is the estimated nonunique equity tweets to which the followers of the 798 accounts (n=6,266,269) can potentially be exposed (mean 0.99, SD 3.89). Linear regression results showed that the number of accounts followed was positively associated with the number of racial equity tweets potentially seen (β=0.06, SE 0.000; *P*<.001). However, since only 309 (38.7%) out of the 798 potential disseminators posted any equity-oriented content, the estimated exposure for more than half (3,490,864/6,266,269, 55.7%) of all followers would be zero CRC racial equity tweets.

To determine which potential disseminator account has the most unique reach into the communities who would otherwise not learn about the topic, we evaluated the portion (%) of each account’s followers for whom they are the only source of racial equity content. In other words, for what portion of their followers does each account category tend to be the only source? The results showed that brokers were substantially more likely to serve that role. For brokers, the average proportion of followers for whom they were the only source of equity tweets is 30%, with the median being 27% (IQR 17%-39%). By contrast, for nonbrokers, the average percentage is 11%, with the median being 10% (IQR 7.4%-14.5%). A linear regression was run using the percentage of followers for whom the source is the unique source as the dependent variable. Brokers, compared with nonbrokers, had a significantly higher proportion of their followers subscribing to them as unique sources of equity content (β=18.9, *P*<.001). These figures do not differ substantially between expert versus nonexperts (β=–0.5, *P*=.90), and including expert as an interaction term does not produce a difference (β=1.1, *P*=.80). The results indicate that broker accounts (regardless of expert or nonexpert status) who tweeted about equity had a unique potential, since they were the only source of this information for a much larger portion of their followers compared with nonbrokers who did so.

## Discussion

### Principal Findings

This study examined CRC content in the context of Twitter, focusing on racial equity–related discussions from CRC equity disseminator accounts on the platform. We analyzed the volume of CRC racial equity tweets overall and by specific content categories (eg, outcome disparity, screening, and narratives) and account types (experts vs nonexperts, brokers vs nonbrokers), and we identified the kinds of accounts that were most likely to uniquely expose their followers to racial equity related information. Findings provide insights into promoting cancer equity through health communication focusing on the role of brokers on social media.

Regarding the volume of CRC racial equity tweets, race and ethnicity was infrequently discussed on Twitter even among potential disseminators (accounting for only 5.8% of the unique tweets). This finding is consistent with previous research on health news stories from newspapers and local TV programs, which also found scant mentions of race or ethnicity [8,9]. Without featuring race and ethnicity–specific health information, people may not pay attention to CRC-related racial inequities. A lack of content tailored to racially and ethnically minoritized groups is concerning in light of the fact that tailored communication could better motivate individual and collective action [14].

While race and ethnicity is not a frequent feature overall in tweets from potential equity disseminators, outcome disparity is the most common content type when race and ethnicity is depicted. Emphasizing the differences in CRC risks and outcomes between racial groups may increase people’s awareness of the disparity gap. However, research also found that when racial health disparity information was presented alone, it might have an adverse impact on the group with higher disease risks (the disadvantaged group), reducing their intention to engage in health behaviors [11-13]. This concern is intensified as the least frequent types of content in racial equity tweets are about strategies that could address CRC outcomes, including screening procedures, prevalence, and benefits. Featuring comparatively high disease risks without also highlighting detailed information about what can be done, how to do it, and the effectiveness of the solutions (eg, CRC screening) could trigger message resistance and behavioral inhibition [12]. Screening prevalence among Black Americans emphasizing progress toward addressing inequities may better motivate screening behavior compared with disparity information in and of itself [13]. The benefits of screening content could also convey the effectiveness of screening, which may serve as behavioral evaluation and response efficacy information both of which are important behavioral antecedents [34,35]. In addition, past research has found that Black Americans were more likely to come across screening-specific information via the web compared with White Americans, which was positively associated with screening behavior [11]. Thus, CRC racial equity tweets may benefit from including more screening-specific information.

However, the overall impact of these tweets may be less than one might assume. Our analysis showed that in line with our concern about the potential for CRC equity messages to reach new audiences, the accounts that talked the most about equity—experts—were less likely to reach unique audiences who would otherwise not learn about this topic. Consistent with network theory, brokers who tweeted about equity were more likely to serve that role since they were the only source of such information for a major portion of their followers. In essence, while experts were more vocal, the more insular nature of their following and networks may make them less capable as disseminators. This suggests that experts or others concerned about the dissemination of equity content might consider allying with accounts that have a greater or unique reach, identifying messages that these far-reaching broker accounts are likely to retweet, rather than simply producing more messages themselves.

### Limitations

The study has several limitations. We identified potential disseminator accounts based on the 7 major CRC racial equity organization accounts with the most followers on Twitter (1500 or more). However, they represent only a part of the many equity-oriented organizations in the United States, and future research may include other related equity organizations with fewer followers on Twitter. Similarly, we only focused on the tweets from these accounts even though other Twitter accounts that are were not in this sample may also post CRC racial equity content. Thus, a user may be exposed to racial equity content, despite not following any of our potential disseminator accounts. Moreover, by focusing on only account followers, our measure of exposure to racial equity tweets might be a more conservative one. Future research could consider other measures of diffusion beyond follower-following relationship, such as retweet networks, for more complex exposure estimation. This paper also did not examine the actual audiences (eg, demographics of different followers) and the impact of racial equity information diffusion on the audiences of such tweets. If members of historically minoritized racial groups do not see the tweets, this can undoubtedly mitigate some of the impact of such communication. Exploring this important research question will be crucial in gaining a more comprehensive understanding of how sharing such messages can lead to desirable outcomes. Last but not least, the official change of Twitter to X in July 2023, despite being irrelevant to the period of this study, means that future research might not be able to study this topic or other health topics on Twitter in the same way due to issues such as limited data access, shifts in user demographics, and changes in technical affordances. With the increasing prominence of platforms featuring long- and short-form video formats such as YouTube or TikTok [36], more research is needed to understand the availability and exposure to racial equity information on these platforms.

### Conclusions

To conclude, the findings from this study highlight the importance of social media accounts that are in a position to diffuse racial equity information produced by equity organizations to otherwise disconnected audiences. Public health officials should encourage these accounts to post more information that focuses on CRC racial equity–related content and tailor their information to the needs of specific racial groups. In doing so, they should emphasize CRC symptoms and details about the screening guidelines/procedures, prevalence, and benefits in their racial equity posts to center and amplify information about CRC detection and prevention.

## Abbreviations

CRC: colorectal cancer

## Data Availability

The datasets generated or analyzed during this study are available from the corresponding author on reasonable request.
